# A Survival-Related Competitive Endogenous RNA Network of Prognostic lncRNAs, miRNAs, and mRNAs in Wilms Tumor

**DOI:** 10.3389/fonc.2021.608433

**Published:** 2021-02-26

**Authors:** HengChen Liu, MingZhao Zhang, ManYu Shi, TingTing Zhang, ZeNan Zhang, QingBo Cui, ShuLong Yang, ZhaoZhu Li

**Affiliations:** Department of Pediatric Surgery, The Second Hospital Affiliated to Harbin Medical University, Harbin, China

**Keywords:** Wilms tumor, target, lncRNA, miRNA, mRNA, competing endogenous RNA/ceRNA

## Abstract

Wilms tumor (WT) commonly occurs in infants and children. We evaluated clinical factors and the expression of multiple RNAs in WT samples in the TARGET database. Eight long non-coding RNAs (lncRNAs; AC079310.1, MYCNOS, LINC00271, AL445228.3, Z84485.1, AC091180.5, AP002518.2, and AC007879.3), two microRNAs (miRNAs; hsa-mir-152 andhsa-mir-181a), and nine messenger RNAs (mRNAs; TCTEX1D4, RNF133, VRK1, CCNE1, HEY1, C10orf71, SPRY1, SPAG11A, and MAGEB18) were screened from differentially expressed RNAs and used to construct predictive survival models. These models showed good prognostic ability and were highly correlated with tumor stage and histological classification. Additionally, survival-related ceRNA network was constructed using 35 RNAs (15 lncRNAs, eight miRNAs, and 12 mRNAs). KEGG pathway analysis suggested the “Wnt signaling pathway” and “Cellular senescence” as the main pathways. In conclusion, we established a multinomial predictive survival model and a survival-related ceRNA network, which provide new potential biomarkers that may improve the prognosis and treatment of WT patients.

## Introduction

Wilms tumor (WT), a renal malignancy originating from the metanephric blastema, is widespread in infants and children (1). WT accounts for 7% of all pediatric malignancies and occurs in one out of every 10,000 children (2). Fortunately, with the development of treatments, the survival rate of children with WT has increased by nearly 60% (3). The International Society of Pediatric Oncology stated that the combination of nephrectomy and chemotherapy significantly improved overall survival (OS) by more than 90% (4). However, 25% of children still have a poor prognosis based on the tumor stage (5). Therefore, clarifying the cellular process involved in WT development and providing prognostic biomarkers are important steps to improving the survival of patients.

In recent years, several studies have suggested non-coding RNAs as key molecules involved in tumorigenesis and tumor progression (6). microRNAs (miRNAs) are non-coding RNAs composed of 18–25 nucleotides that can negatively regulate gene expression (7). By contrast, long non-coding RNAs (lncRNAs) are more than 200 nucleotides in length and they adjust the biological behavior of tumors through competing endogenous RNAs (ceRNAs) (8). It is suggested that there is a complex regulatory network among lncRNAs, miRNAs, and messenger RNAs (mRNAs). lncRNAs competitively inhibit the function of miRNAs by acting as a sponge, thus indirectly disrupting mRNA expression and ultimately affecting gene expression (9). In general, the instability of a ceRNA network may induce tumorigenesis (10, 11). Several studies have suggested that a ceRNA network can be used as a prognostic biomarker for WT, but did not actually describe the effect of a ceRNA network in WT (12, 13). Therefore, the establishment of a ceRNA network related to the survival of WT patients is of great significance for judging the prognosis of patients. By understanding the role of various RNAs in tumorigenesis, we can find potential targets to improve the prognosis of WT.

In this study, we explored the ability of multiple RNAs to prognosticate WT as a whole and established RNA models that can be used to predict survival. In addition, we established a survival-related ceRNA network and tried to understand its molecular mechanism through functional enrichment. Finally, we provided new potential biological biomarkers to improve the prognosis and treatment of WT.

## Materials and Methods

### Data Collection and Processing

Clinical and RNA sequencing data of all patients were obtained from the Therapeutically Applicable Research to Generate Effective Treatments (TARGET) database, which can be downloaded from The Cancer Genome Atlas (TCGA) portal (https://portal.gdc.cancer.gov/; Data Release 25.0; release time: July 22, 2020). This study met the requirements for using TCGA database and did not require the approval of an ethics committee. We selected only the sequencing data from primary solid tumors for analysis. The mRNA and lncRNA sequencing data included 125 primary WT samples and six normal samples. The miRNA sequencing data included 127 primary WT samples and six normal samples. The clinical data of the 128 patients included in this study are shown in **Table 1**.

**Table 1:** Corresponding Clinical Features of 128 Patients With Wilms Tumor.

Items	Patients, N = 128	
	N	%
Age		
<5	81	63.28125
≥5	47	36.71875
Gender		
Male	54	42.1875
Female	74	57.8125
Race		
White	95	74.21875
Non-White	33	25.78125
Tumor stage		
Stage I/II	66	51.5625
Stage III/IV	62	48.4375
Histologic classification		
FHWT	84	65.625
DAWT	44	34.375
Survival status		
Alive	76	59.375
Dead	52	40.625

### Identification of Differentially Expressed RNAs

The edgeR package in the R 4.0.2 software was used to analyze differentially expressed lncRNAs (DElncRNAs), differentially expressed miRNAs (DEmiRNAs), and differentially expressed mRNAs (DEmRNAs) between the WT and normal samples. The cutoff value for differentially expressed RNAs (DERNAs) was |log2 fold-change (FC)| >1 and false discovery rate (FDR) <0.05. Visualization of DERNAs was performed using ggplot2 package in R software.

### Survival Analysis

dentification of survival-associated RNAs was performed *via* univariate Cox regression analyses in the R software. Significantly correlated survival-associated RNAs (P < 0.003) were chosen for multivariate Cox regression analysis and to establish predictive survival models. Prognosis index (PI) = (expression_gene1_ × *β*_gene1_) + (expression_gene2_ × *β*_gene2_) +… + (expression_genen_ × *β*_genen_). Patients were divided into two groups based on the median PI. Survival prognosis of the two groups was compared by Kaplan–Meier analysis. The receiver operating characteristic (ROC) curve for evaluating the predictive ability of the model was depicted through R software.

### Protein–Protein Interaction Network Construction

The Search Tool for the Retrieval of Interacting Genes (STRING; http://string-db.org) database was used to obtain PPI data of the significant survival-associated mRNAs (P < 0.003). Establishment of the PPI network was performed using the Cytoscape software.

### Construction of the ceRNA Network

Survival-associated RNAs were used to construct ceRNA networks. First, the potential miRNAs interacting with lncRNAs were screened according to the miRcode (http://www.mircode.org/) database. Targeted mRNAs were identified using the miRTarBase (http://mirtarbase.cuhk.edu.cn/), miRDB (http://www.mirdb.org/), and TargetScan (http://www.targetscan.org/) databases. Finally, establishment of the lncRNA-miRNA-mRNA interaction ceRNA network was performed using the Cytoscape software.

### Functional Enrichment Analysis

Gene Ontology (GO) and Kyoto Encyclopedia of Genes and Genomes (KEGG) pathway enrichment analyses of mRNAs in the ceRNA network were conducted using KOBAS 3.0 (http://kobas.cbi.pku.edu.cn/kobas3/). Visualization of the enrichment analyses was conducted using the R software.

### Statistical Analysis

The correlation of RNAs with clinical characteristics was analyzed by rank sum test. Differences between survival curves were analyzed by log-rank test. R 4.0.2, Cytoscape v3.7.2, and GraphPad Prism 8 were used for plotting. SPSS 24 was used for statistical analysis. P < 0.05 was considered statistically significant.

## Results

### Identification of DElncRNAs, DEmiRNAs, and DEmRNAs

We downloaded RNA sequencing data of 128 WT patients from the database and screened multiple DERNAs separately. A total of 10,585 DERNAs were screened, including 3,219 DElncRNAs (1,664 upregulated and 1,555 downregulated), 236 DEmRNAs (153 upregulated and 83 downregulated), and 7,130 DEmRNAs (3,762 upregulated and 3,368 downregulated). Finally, we visualized multiple DERNAs by heat and volcano maps (**Figure S1**).

### Identification of Survival-Associated RNAs in WT

**Figure 1:**
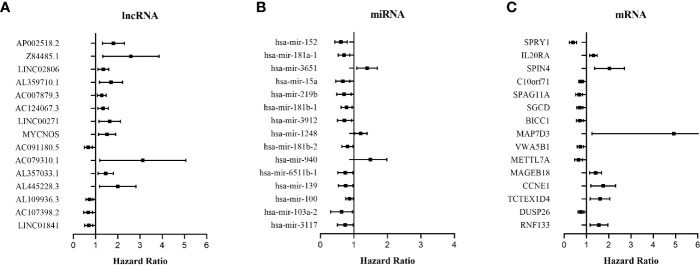
Forest plots of the hazard ratios (HR) of the survival-associated RNAs in WT. **(A)** HR of top 15 survival-associated lncRNAs. **(B)** HR of top 15 survival-associated miRNAs. **(C)** HR of top 15 survival-associated mRNAs. HR <1 indicates the protective RNAs, and HR >1 indicates the risk RNAs.

**Figure 2:**
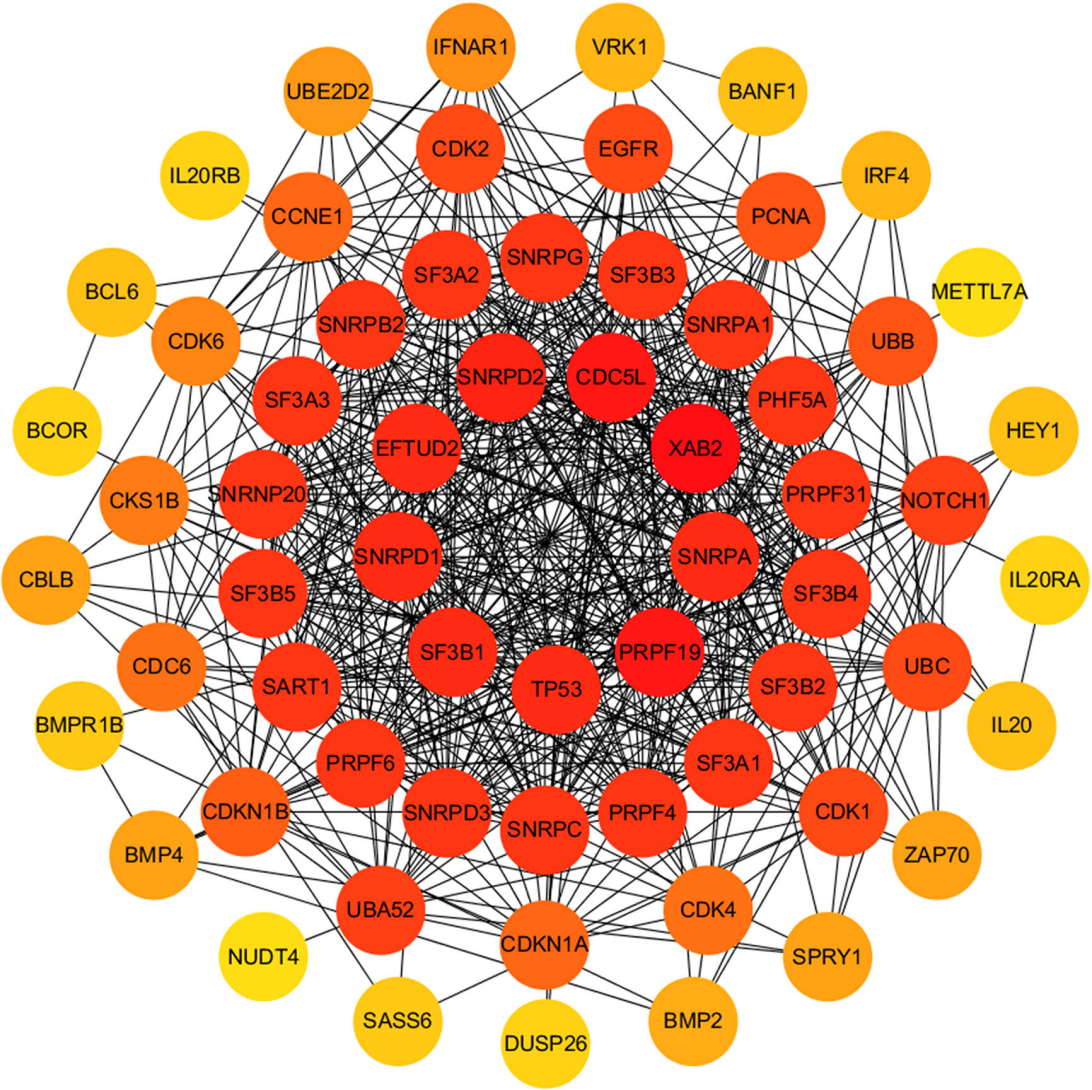
PPI network of significant survival-associated mRNAs by Cytoscape. The brightness of the circle represents the degree of connection. The red circles are hub genes in the networks.

The relationship between DERNAs and survival was assessed by univariate Cox regression analysis. RNAs with P <0.05 were selected as survival-associated RNAs, yielding a total of 696 survival-associated RNAs (199 lncRNAs, 17 miRNAs, and 480 mRNAs). The top 15 survival-associated RNAs are shown in **Figure 1**. The gene networks of strongly correlated survival-associated mRNAs (P < 0.003) were constructed by STRING (**Figure 2**). The hub genes, including XAB2, SNRPA, PRPF19, and TP53, are shown in the PPI network.

### Establishment of Predictive Survival Models

RNAs with strong correlation were selected by univariate Cox regression analysis (P < 0.003), and then multivariate Cox regression analysis was used to analyze survival-associated RNAs with strong correlation. Finally, a total of eight lncRNAs (AC079310.1, MYCNOS, LINC00271, AL445228.3, Z84485.1, AC091180.5, AP002518.2, and AC007879.3), two miRNAs (hsa-mir-152 and hsa-mir-181a), and nine mRNAs (TCTEX1D4, RNF133, VRK1, CCNE1, HEY1, C10orf71, SPRY1, SPAG11A, and MAGEB18) were identified. Subsequently, the predictive survival models were built. PI_lncRNA_ = (0.76777 × AC079310.1 expression) + (0.28668 × MYCNOS expression) + (0.53416 × LINC00271 expression) + (0.64448 × AL445228.3 expression) + (0.45112 × Z84485.1 expression) + (− −0.35751 × AC091180.5 expression) + (0.53728 × AP002518.2 expression) + (0.14176 × AC007879.3 expression). PI_miRNA_ = (−0.3952 × hsa-mir-152 expression) + (−0.2490 × hsa-mir-181a expression). PI_mRNA_ = (0.52018 × TCTEX1D4 expression) + (0.24841 × RNF133 expression) + (0.48661 × VRK1 expression) + (0.40846 × CCNE1 expression) + (−0.43723 × HEY1 expression) + (−0.17615 × C10orf71 expression) + (-0.32992 × SPRY1 expression) + (−0.25891 × SPAG11A expression) + (0.29755 × MAGEB18 expression).

**Figure 3:**
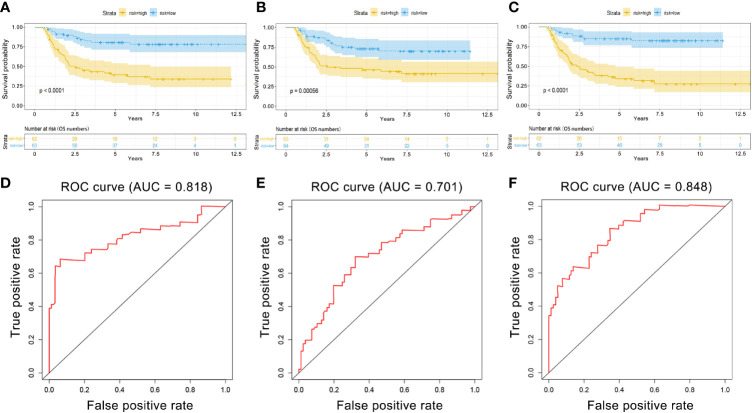
Kaplan–Meier (K–M) and ROC curves for PI in WT patients. **(A)** K–M survival curves between high-risk and low-risk groups based on lncRNA model. **(B)** K–M survival curves between high-risk and low-risk groups based on miRNA model. **(C)** K–M survival curves between high-risk and low-risk groups based on mRNA model. **(D)** Time-dependent ROC curves analysis for survival prediction by PIlncRNA. **(E)** Time-dependent ROC curves analysis for survival prediction by PImiRNA. **(F)** Time-dependent ROC curves analysis for survival prediction by PImRNA.

**Figure 4:**
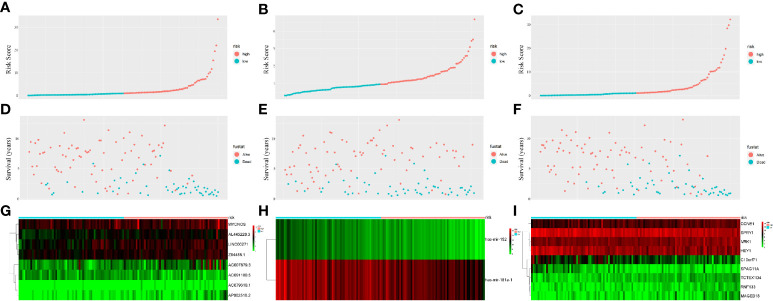
Prognostic classifier analyses in distinguishing patients into low-risk and high-risk groups. **(A)** The lncRNA-based risk score distribution of each patient. **(B)** The miRNA-based risk score distribution of each patient. **(C)** The mRNA-based risk score distribution of each patient. **(D)** The lncRNA-based survival status of each patient in two groups. **(E)** The miRNA-based survival status of each patient in two groups. **(F)** The mRNA-based survival status of each patient in two groups. **(G)** Heatmap of lncRNAs expression between two groups. **(H)** Heatmap of miRNAs expression between two groups. **(I)** Heatmap of mRNAs expression between two groups.

PI values were calculated for each patient and divided into two groups. We found that among the three groups of RNAs, the low-risk group had better survival as determined by Kaplan–Meier analysis (**Figures 3A–C**). The ability of the models to predict 3-year survival was evaluated by drawing the ROC curve. The areas under the curves (AUCs) of the three groups were 0.818, 0.701, and 0.848, respectively (**Figures 3D–F**). These results suggest that the three groups of modules have great potential for predicting the clinical prognosis of WT. **Figure 4** shows the risk scores, survival status, and RNA expression profiles in each group.

**Figure 5:**
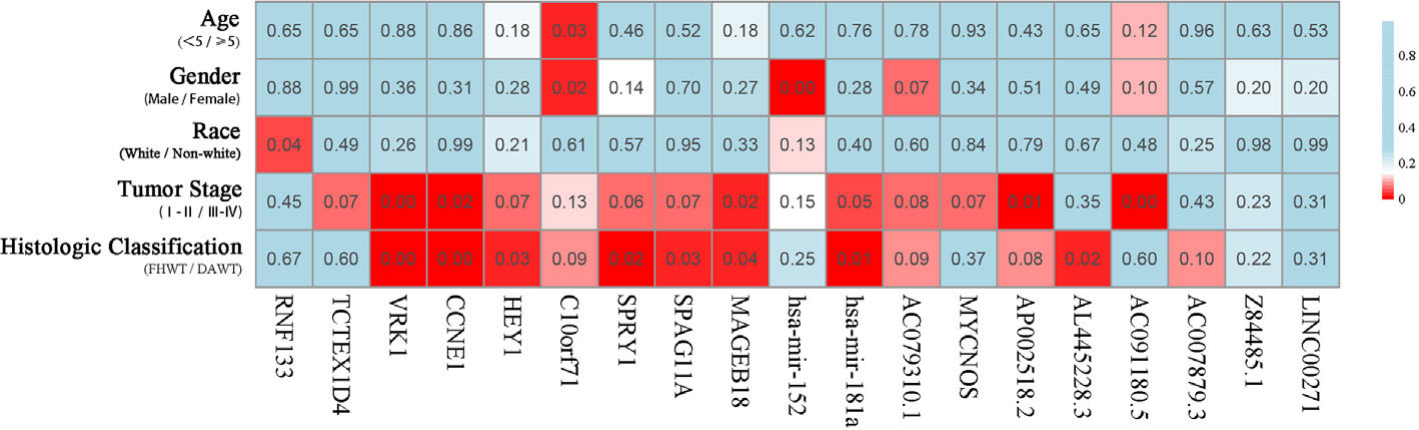
Clinical significance of prognostic RNAs in WT. Correlations between prognostic RNAs and clinical factors. The numbers and brightness of each cell indicate the P-value.

The correlation of these RNAs with other clinical characteristics was assessed by rank sum test. Clinical characteristics included age (<5/≥5), gender (male/female), race (white/non-white), tumor stage (I–II/III–IV), and pathological classification (FHWT/DAWT). We found that the RNAs in the models were significantly correlated with tumor stage and histological classification (**Figure 5**). This implies that these RNAs can be used as potential indicators to judge the degree of WT tumor development. Next, we identified the relationship between clinical characteristics and OS. Multivariate Cox regression analysis suggested that tumor stage and risk level directly affected tumor prognosis (**Table 2**).

**Table 2:** Univariate and multivariate Cox regression analyses of overall survival.

Overall survival	Univariate analysis			Multivariate analysis		
	HR	95%CI	P-value	HR	95%CI	P-value
Age (<5/≥5)	0.704	0.386–1.284	0.252			
Gender (Male/Female)	0.591	0.343–1.019	0.058			
Race (White/Non-white)	1.122	0.607–2.071	0.714			
Tumor stage (I-II/III-IV)	0.999	1.792–5.840	0.998			
lncRNA cohort				3.504	1.882–6.524	<0.001
miRNA cohort				3.105	1.717–5.615	<0.001
mRNA cohort				2.454	1.307–4.608	0.005
Histologic classification (FHWT/DAWT)	1.196	0.714–2.209	0.529			
LncRNA signature (Low group/High group)	4.502	2.346–8.637	<0.001	4.822	2.472–9.403	<0.001
miRNA signature (Low group/High group)	2.673	1.495–4.777	0.001	2.498	1.394–4.474	0.002
mRNA signature (Low group/High group)	6.380	3.173–12.830	<0.001	4.883	2.381–10.011	<0.001

### Construction of a Survival-Related ceRNA Network in WT

**Figure 6:**
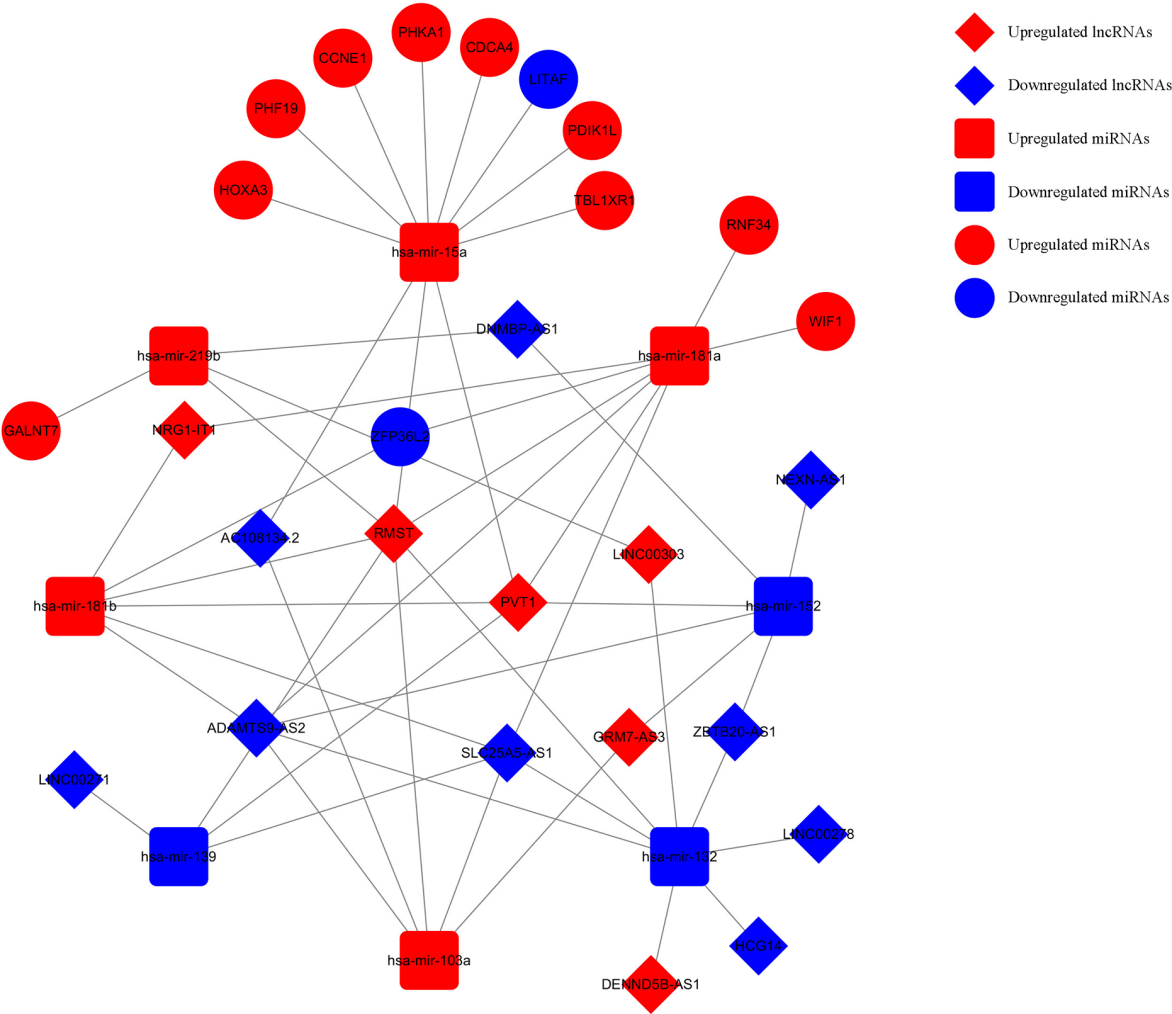
Survival-related ceRNA network in WT. The red diamonds indicate upregulated lncRNAs, and blue diamonds indicate downregulated lncRNAs. The red rectangles indicate upregulated miRNAs, and blue rectangles indicate downregulated miRNAs. The red circles diamonds indicate upregulated mRNAs, and blue circles diamonds indicate downregulated mRNAs.

**Figure 7:**
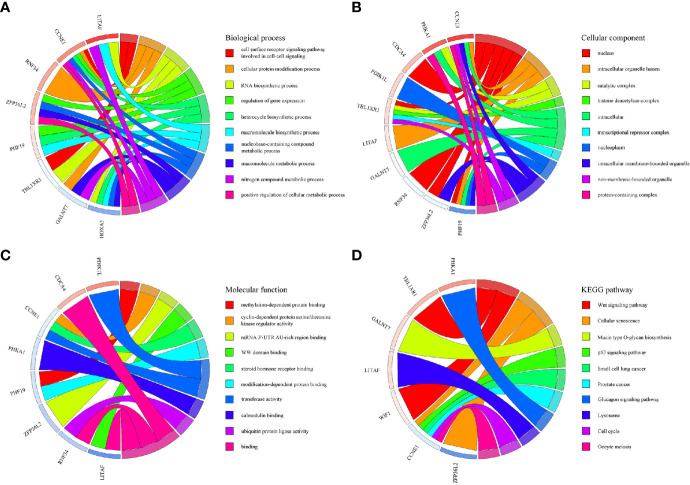
GO and KEGG pathway enrichment analyses of genes included in the ceRNA network. **(A)** Biological process. **(B)** Cellular component. **(C)** Molecular function. **(D)** KEGG pathways.

Based on the survival-associated RNAs, 39 pairs of lncRNA–miRNA and 13 pairs of miRNA–mRNAs were detected from the databases. Subsequently, a ceRNA network containing 15 lncRNAs, eight miRNAs, and 12 mRNAs was constructed (**Figure 6**). Many of these RNAs have been extensively studied as cancer-related molecules, such as miR-181a, CCNE1, and WIF1. Next, we further studied the molecular function of mRNAs in the ceRNA network. A total of 99 functional enrichment terms (68 biological processes, 13 cellular components, and 18 molecular functions) from the GO analysis and 15 KEGG pathways were observed. The biological processes were mainly enriched in “cell surface receptor signaling pathway involved in cell-cell signaling”; the cellular components were mainly enriched in “nucleus”; and the molecular function was mainly enriched in “binding”. The KEGG analysis suggested that the “Wnt signaling pathway” and “Cellular senescence” were the main pathways (**Figure 7**).

## Discussion

WT is a common pediatric tumor and poor prognosis is the main factor affecting long-term survival of patients (14). However, the exact molecular mechanism of WT is still unclear. The advancement in sequencing technology and the proposed ceRNA hypothesis provide a new perspective for the study of tumorigenesis and tumor progression (9). In the present study, we screened out survival-associated RNAs in the TARGET database and studied molecular events associated with WT. In particular, we proposed a novel survival-related ceRNA network that provides a new dimension for predicting the prognosis of WT patients.

Among all the DERNAs, we screened a total of 696 survival-related RNAs, including 199 lncRNAs, 14 miRNAs, and 480 mRNAs. mRNAs are key to the realization of molecular functions. PPI network analysis was carried out to identify the hub genes. We found that XAB2, SNRPA, PRPF19, and TP53 had a higher degree of connection among all genes. It is well-known that p53 is a tumor suppressor gene, and some studies have confirmed that TP53 gene mutation worsens the prognosis of WT (15). The other hub genes also affect the progress of other tumors (16, 17), suggesting that these hub genes may also affect the development of WT.

Most recent studies on WT have focused on the search for a single RNA that may influence prognosis. Zong et al. (18) found that miR-30d expression in WT tissues was significantly lower than that in normal tissues. Further animal experiments showed that miR-30d mimic significantly inhibited tumor growth. In addition, overexpression of SOX4 could reverse the effect of mir-30d on WT cells. Bao et al. (19) found that the expression of mirna-203a was low in WT tumor tissues, and that its expression level was directly related to the prognosis of WT. Knockout of miR-203a significantly enhanced the invasiveness of WT cells. Luciferase analysis confirmed that miR-203a targeted JAG1 to exert biological functions. Wang et al. (20) found that overexpression of miR-613 inhibited the G0/G1 phase transition of WT cells and hindered the expansion ability of WT cells. There is also a subset of studies evaluating the relationship between single RNA and OS. Tang et al. (12) predicted 32 DERNAs to be possibly associated with OS in WT patients through Kaplan-Meier analysis, and Zhang et al. (13) predicted 61 DERNAs to be possibly associated with OS by Kaplan-Meier analysis.

However, the development of WT is influenced by multifactorial causes, indicating that we should analyze prognostic markers at a broader level. We established a multinomial predictive survival model based on survival-associated RNAs by multivariate Cox analysis, which could suggest prognostic survival of WT patients. An AUC value >0.70 denotes excellent model performance. The AUC values of the three models were all greater than 0.7 in our study (0.818, 0.701, and 0.848). Gong et al. (21) established a 5-miRNA model for the prognosis of WT patients. The AUC value of their model was 0.767, which was higher than that of the miRNA model in this experiment, but smaller than those of the other two models in this experiment. In addition, compared with other experiments, this experiment only selected the sequencing data of primary tumor tissues rather than the sequencing data of all tumors including metastatic tumors. Further, we divided the patients into high-risk and low-risk groups according to PI value, and Kaplan–Meier analysis showed that there was a significant difference in survival between the two groups. Visualizing the relationship among survival time, PI score, and grouping intuitively showed the poor survival of high-risk patients. These results indicate that these three RNA models have good specificity and sensitivity in predicting the survival of WT patients. Notably, we found that these predictive RNAs were significantly associated with tumor stage and histological classification, which means that PI may have great value in the prognosis of WT patients. Multivariate Cox regression analysis also suggested that risk level can predict the prognosis of WT patients.

According to the establishment of the predictive survival models, we found some biomarkers that may have important clinical significance. lncRNAs participate in multiple cellular activities and have been shown to regulate tumorigenesis and metastasis (22, 23). miRNAs, as a bridge between lncRNAs and mRNAs, participate in tumor development. MYCNOS promotes the invasion and metastasis of neuroblastoma and rhabdomyosarcoma by regulating MYCN protein (24, 25) and may participate in the development of WT (26). miR-152 targeting DNMT1 inhibits the development of endometrial cancer (27), glioblastoma (28), and lymphomas (29). miR-181a mediates the Wnt/*β*-catenin pathway to accelerate the progression of colorectal cancer (30), oral squamous cell carcinoma (31), and acute lymphoblastic leukemia (32). The expression of VRK1 directly affects the proliferation of breast cancer and liver cancer (33). CircAGFG1 promotes triple-negative breast cancer progression through the circAGFG1/miR-195/CCNE1 pathway (34). Precancerous lesions in squamous cell carcinoma depend on upregulation of the NOTCH4-HEY1 pathway (35). The HGF-mediated c-Met/FRA1/HEY1 cascade may be the key to inducing the transition from cirrhosis to hepatocellular carcinoma (36). miRNA-21 promotes proliferation of human glioma cells through the PI3K/AKT/SPRY1 pathway (37). The expression of MAGEB18 affects cell proliferation and apoptosis in melanoma (38). However, the effects of the aforementioned RNAs on WT progression have not been reported at present. In addition, it remains unknown whether AC079310.1, LINC00271, AL445228.3, Z84485.1, AC091180.5, AP002518.2, AC007879.3, TCTEX1D4, RNF133, CCNE1, C10orf71, and SPAG11A can influence the development of tumors. These RNAs provide a potential direction for future investigations on WT.

The ceRNA hypothesis explains the complicated RNA molecular regulation mechanisms *via* construction of a lncRNA–miRNA–mRNA network (9). Most studies on the molecular regulation mechanism of WT constructed a network based on DERNAs (12, 13). We proposed a novel survival-related ceRNA network that provided a new dimension for predicting the prognosis of patients with WT. Many cancer-related molecules are included in this network, such as miR-181a, CCNE1, and WIF1. Recent studies have determined that some RNAs in the ceRNA network affect the prognosis of WT patients. For example, miR-15a targeted by SNHG6 (39), cyclin CCNE1 regulated by tumor suppressor gene WWOX (40), and Wnt signaling pathway inhibited by WIF1 (41) all affect tumor prognosis. In view of the fact that mRNA is the executor of ceRNA network function, GO and KEGG analyses were conducted to gain insight into molecular mechanisms. The GO analysis suggested that the cell surface receptor signaling pathway is the main mechanism by which the ceRNA network affects the prognosis of WT patients. KEGG analysis suggested that the “Wnt signaling pathway” and “Cellular senescence” were the main enrichment pathways. Recently, the Wnt/*β*-catenin signaling pathway was demonstrated to regulate the occurrence and development of WT, and Wnt-targeted agents may have great potential in the treatment of WT (42, 43).

Our study had several limitations. First, due to the scarcity of patients, we only obtained RNA sequencing data from the TARGET database; thus, we could not perform multicenter validation. Second, all the data in this study are from the same pathological tissue and lacked certain accuracy for highly heterogeneous WT. In the future, we will use single-cell sequencing to detect the expression RNAs in multiple WT samples from the same patient to improve the accuracy of the results. Finally, some new RNAs that may be involved in WT progression need further study.

To conclude, we established a multinomial predictive survival model, which has a good application prospect in future clinical practice. It is expected to improve the long-term prognosis of WT patients by screening high-risk patients through sequencing results and strengthening the personalized treatment. Meanwhile, we describe a survival-related ceRNA network, which provides some new potential prognostic indicators and therapeutic targets for improving the prognosis of WT patients.

## Data Availability Statement

The original contributions presented in the study are included in the article/**Supplementary Material**. Further inquiries can be directed to the corresponding author.

## Author Contributions

HL and ZL designed the study. HL, MZ, and MS performed the data collection. HL, MZ, and TZ analyzed the data. HL, ZZ, and SY wrote the manuscript. HL and QC revised the manuscript. All authors contributed to the article and approved the submitted version.

## Funding

This study was supported by the National Natural Science Foundation of China (81871837, 81572117) and the Specialized Research Fund for Doctoral Program of Higher Education of China (20132307110007).

## Conflict of Interest

The authors declare that the research was conducted in the absence of any commercial or financial relationships that could be construed as a potential conflict of interest.

The handling editor declared a shared affiliation, though no other collaboration, with the authors.
