# Supplementary figures and images for: A Survival-Related Competitive Endogenous RNA Network of Prognostic lncRNAs, miRNAs, and mRNAs in Wilms Tumor
Source: Front Oncol. 2021 Feb 26;11:608433. doi: 10.3389/fonc.2021.608433 (PMC7953909; doi:10.3389/fonc.2021.608433)

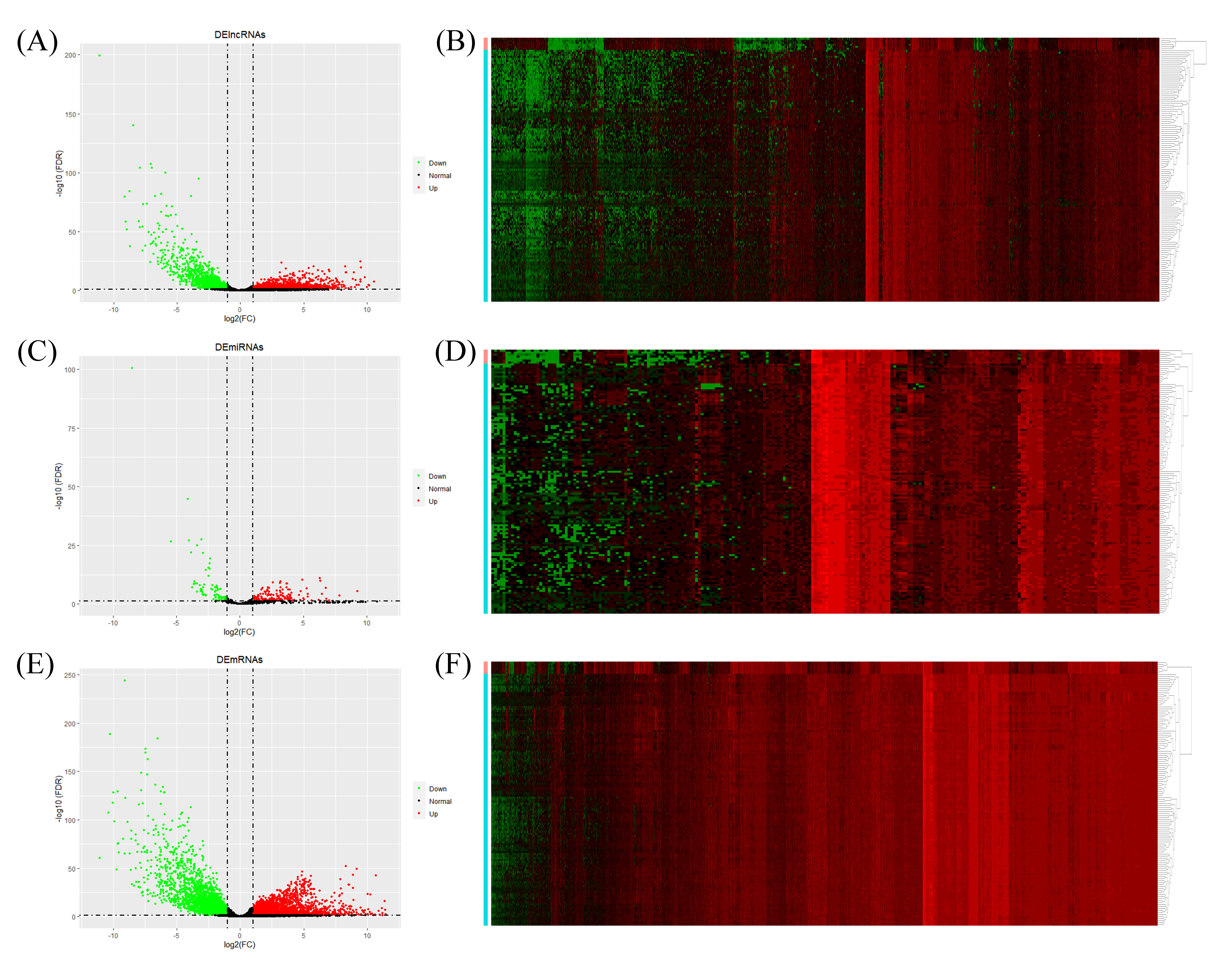

Supplement: Supplementary file 1 [file Image_1.jpeg]
